# Stimulator of interferon genes defends against bacterial infection *via* IKKβ-mediated Relish activation in shrimp

**DOI:** 10.3389/fimmu.2022.977327

**Published:** 2022-08-19

**Authors:** Haoyang Li, Qinyao Li, Sheng Wang, Jianguo He, Chaozheng Li

**Affiliations:** ^1^ State Key Laboratory of Biocontrol, School of Marine Sciences, Sun Yat-sen University, Guangzhou, China; ^2^ Southern Marine Science and Engineering Guangdong Laboratory (Zhuhai), Zhuhai, China; ^3^ Guangdong Provincial Key Laboratory of Marine Resources and Coastal Engineering/Guangdong Provincial Key Laboratory for Aquatic Economic Animals, Sun Yat-sen University, Guangzhou, China; ^4^ Guangdong Laboratory for Lingnan Modern Agriculture, Maoming, China; ^5^ China-ASEAN Belt and Road Joint Laboratory on Marine Aquaculture Technology, Zhuhai, China

**Keywords:** Litopenaeus vannamei, STING, IKKβ, relish, vibrio parahaemolyticus

## Abstract

Stimulator of interferon genes (STING) is crucial for the innate immune to defend against pathogenic infections. Our previous study showed that a STING homolog from Litopenaeus vannamei (LvSTING) was involved in antibacterial response *via* regulating antimicrobial peptides (AMPs). Nevertheless, how LvSTING induces AMPs expression to inhibit bacterial infection remains unknown. Herein, we revealed that the existence of a STING–IKKβ–Relish–AMPs axis in shrimp that was essential for opposing to Vibrio parahaemolyticus invasion. We observed that LvRelish was essential for host defense against V. parahaemolyticus infection *via* inducing several AMPs, such as LvALF1, LvCRU1, LvLYZ1 and LvPEN4. Knockdown of LvSTING or LvIKKβ *in vivo* led to the attenuated phosphorylation and diminished nuclear translocation of LvRelish, as well as the impaired expression levels of LvRelish-regulated AMPs. Accordingly, shrimps with knockdown of LvSTING or LvIKKβ or both were vulnerable to V. parahaemolyticus infection. Finally, LvSTING could recruit LvRelish and LvIKKβ to form a complex, which synergistically induced the promoter activity of several AMPs *in vitro*. Taken together, our results demonstrated that the shrimp STING–IKKβ–Relish–AMPs axis played a critical role in the defense against bacterial infection, and provided some insights into the development of disease prevention strategies in shrimp culture.

## Introduction

Shrimp farming is the important part of fishing industry, and the economic value of shrimp aquaculture has increased at an annual growth rate of 7.6% from ~26.1 billion dollars in 2013 to ~40.5 billion dollars in 2019 (1). Nevertheless, recent frequent outbreaks of bacterial diseases have resulted in tremendous economic losses (2). Vibrio species, the main pathogens causing bacterial diseases, have been frequently detected in penaeid shrimp culture ponds with at least 14 species implicated (e.g. *Vibrio parahaemolyticus*, *Vibrio harveyi* and *Vibrio alginolyticus*) (3). The white feces syndrome (WFS), a worldwide severe non-infectious shrimp disease, has been related to the Vibrio overrepresented in host intestine (4). Besides, *V. parahaemolyticus* that containing a virulence plasmid to encode a binary toxin PirA and PirB, is considered to cause acute hepatopancreatic necrosis disease (AHPND) (2). Shrimp innate immunity has become the focus of increased research in an effort to create disease prevention techniques due to the threat of bacterial infections.

Stimulator of interferon genes (STING) is the vital protein implicated in a wide range of innate immune responses to viral, bacterial, and parasite infections (5). The cyclic GMP-AMP synthase (cGAS) senses the DNA segments from pathogens and generates the second messenger cGAMP binding to STING. The activated STING promotes the activation of interferon (IFN) regulatory factor 3 (IRF3) and NF-κB transcription factors, leading to the production of type I interferons and inflammatory cytokines. Due to the loss of the zinc-ribbon domain, most invertebrate cGAS homologs have been considered to have no function as a DNA sensor (6). Therefore, invertebrate STING mediated signal pathways appear to have different activation mechanisms. In *Nematostella vectensis*, the cGAS (NvcGAS) activity of CDN synthesis can be activated by some unknown ligands instead of DNA, and NvSTING can recognize the 2’3’-cGAMP produced by NvcGAS (7). In *Crassostrea gigas*, there is a conservative STING-dependent signaling performed with STING binding to 2’3’-cGAMP (8). *Drosophila melanogaster* cGAS-like protein DmcGLR1 can sense viral dsRNA and produce 2’3’-cGAMP, while DmGLR2 can respond to virus infection and produce both 2’3’-cGAMP and 3’2’-cGAMP (9). DmSTING binds to 2’3’-cGAMP and 3’2’-cGAMP, then triggers the STING–IKKβ–Relish signaling axis to oppose virus infection in flies (10–12). A shrimp study shows that *Litopenaeus vannamei* cGAS homolog (LvMab21cp) is unable to increase dsDNA-activated LvSTING-dependent I IFN-β and IFN-ω production in HEK293T cells, because it lacks the typical structures for DNA sensing and cGAMP production (13). And *L. vannamei* STING (LvSTING) can react to *V. parahaemolyticus* infection by inducing LvPEN4, one kind of antimicrobial peptides, to protect shrimp from vibriosis (14). Regardless, how LvSTING regulates antimicrobial peptides (AMPs) remains unrevealed.

Herein, we established the shrimp STING–IKKβ–Relish–AMPs axis that conferred host defense against *V. parahaemolyticus* infection. These data provided several new insights into shrimp bacterial disease control.

## Materials and methods

### Shrimp and *V. parahaemolyticus*

Shrimp weighing an average of roughly 5 g were obtained from Guangzhou, Guangdong Province, China. The shrimp were cultured for 3 days in aerated seawater (30‰ salinity, 25°C) and fed a commercial food (HAID Group) three times daily prior to the experiment. *V. parahaemolyticus* (isolated from a diseased shrimp by our lab) were cultured in Luria broth (LB) medium overnight at a temperature of 37°C (4). The cultured *V. parahaemolyticus* were quantified through counting the microbial colony-forming units (CFUs) per milliliter on LB agar plates. The final injection concentration of *V. parahaemolyticus* was controlled to approximately 1 × 105 CFU/50 μl (15).

### Plasmid construction

The open reading frame (ORF) of LvSTING (Genbank accession KY490589.1) was cloned into pAc5.1-HA vectors (16) to generate pAc-LvSTING-HA. The ORF of LvIKKβ (Genbank accession JN180642) was constructed into pAc5.1-FLAG vector (17) for expressing FLAG-tagged LvIKKβ protein. The ORF of LvRelish (Genbank accession EF432734) was constructed into pAc5.1-V5 vector (Invitrogen, Cat No. V4110-20, USA) for expressing V5-tagged LvRelish protein. GFP sequence was constructed into pAc5.1-HA vector to express HA-tagged GFP. The promoter sequences of *L. vannamei* anti-LPS-factor 1 (LvALF1, Genbank accession EW713395), Crustin 1 (LvCRU1, Genbank accession AF430071.1), Lysozyme 1 (LvLYZ1, Genbank accession JN039375.1) and (LvPEN4, Genbank accession DQ206402) were cloned into pGL3-basic vector (Promega, Cat No. E1751, USA). Primer sequences were listed in **Table 1**.

**Table 1:** Primers used in this study.

Protein expression	
LvRelish-F	AGGGGTACCATGGTGAGAGGTGACAGAGGTGG
LvRelish-R	ACCGGGCCCCGCCTGGTCCAGTACAGCTACACATTCC
LvIKKβ-F	CCGCTCGAGATGGCAGCAGCAGAAGACCGTC
LvIKKβ-R	GCTCTAGACAAGGAAGTTTCAACTGCCTTCTTAT
LvSTING-F	AGGGGTACCATGAAGGGAGACGAGCTGGTC
LvSTING-R	AACGGGCCCTCAGCAAAACAAAAGAGATTCTGCCGCT
GFP-F	AGGGGTACCATGGTGAGCAAGGGCGAGGAGCTGT
GFP-R	AACGGGCCCTCACTTGTACAGCTCGTCCATGCCGAGA
Dual luciferase assay	
LvALF1-F	GGGGTACCCTTGATTAGCCGATCCCAGAC
LvALF1-R	GGAGATCTACTACAGAGCTGACCAGCACCC
LvLYZ1-F	GGGGTACCCTATGGTGAATGCCACCGGGCAG
LvLYZ1-R	GGAGATCTGGTTCCGAAGTGTAAGTTGCTTG
LvCRU1-F	GGGGTACCCTGGAAAATACCAGGTGTTGATG
LvCRU1-R	GGAGATCTGTTGCCTCCAGTACAAGCTAGTG
LvPEN4-F	GGGGTACCACATGCAGATACAGATACATATATTCATATT
LvPEN4-R	GGAAGATCTGCGGACGCAGGAGGCAAC
Quantitative PCR (qPCR)	
LvEF-1α-F	TATGCTCCTTTTGGACGTTTTGC
LvEF-1α-R	CCTTTTCTGCGGCCTTGGTAG
LvRelish-F	AACACCTCCTCCTTCACCC
LvRelish-R	GGTCTCAGTGCCAGAGTAGGT
LvIKKβ-F	ACCACACTTTCCACCTTTGG
LvIKKβ-R	TCCCGATGAAGGAAGAACAC
LvSTING-F	CTCAGACACTCGTGGGAGGC
LvSTING-R	CCTGTGCTGCTGTTCGAAGG
LvLYZ1-F	TACGCGACCGATTACTGGCTAC
LvLYZ1-R	AGTCTTTGCTGCGACCACATTC
LvALF1-F	TTACTTCAATGGCAGGATGTGG
LvALF1-R	GTCCTCCGTGATGAGATTACTCTG
LvCRU1-F	GTAGGTGTTGGTGGTGGTTTC
LvCRU1-R	CTCGCAGCAGTAGGCTTGAC
LvPEN4-F	GTTACCCAAACCATCCCGAC
LvPEN4-R	CAGACTATCCTCTGTGACAACAATC
Vpa-16s-F	GGTGTAGCGGTGAAATGCGTAG
Vpa-16s-R	CCACAACCTCCAAGTAGACATCG
dsRNA templates amplification	
GFP-F	CGACGTAAACGGCCACAAGTT
GFP-R	ATGGGGGTGTTCTGCTGGTAG
GFP-T7-F	GGATCCTAATACGACTCACTATAGGCGACGTAAACGGCCACAAGTT
GFP-T7-R	GGATCCTAATACGACTCACTATAGGATGGGGGTGTTCTGCTGGTAG
LvRelish-F	TTGAGTTGGATGAGAATGATCGGGAAGT
LvRelish -R	CCTGAAGAAGGCTGTTATTGATGGTGGT
LvRelish -T7-F	GGATCCTAATACGACTCACTATAGGTTGAGTTGGATGAGAATGATCGGGAAGT
LvRelish -T7-R	GGATCCTAATACGACTCACTATAGGCCTGAAGAAGGCTGTTATTGATGGTGGT
LvIKKβ-F	GCTGCTGTCCGTTCCTGC
LvIKKβ-R	TTTCTCCATTGCGACCTTCA
LvIKKβ-T7-F	GGATCCTAATACGACTCACTATAGGGCTGCTGTCCGTTCCTGC
LvIKKβ-T7-R	GGATCCTAATACGACTCACTATAGGTTTCTCCATTGCGACCTTCA
LvSTING-F	GGCCATCGGCTACTACGTC
LvSTING -R	ATCCCGTACCATCGATTTCCAT
LvSTING -T7-F	GGATCCTAATACGACTCACTATAGGGGCCATCGGCTACTACGTC
LvSTING -T7-R	GGATCCTAATACGACTCACTATAGGATCCCGTACCATCGATTTCCAT

### Co-immunoprecipitation and western blot

Co-immunoprecipitation (Co-IP) assays were performed to investigate the interaction of endogenous proteins ectopic expressed proteins in cells or in shrimp hemocytes. In *Drosophila* S2 cells, the plasmids pAc-LvRelish-V5 or pAc-LvIKKβ-FLAG were co-transfected with pAc-LvSTING-HA. The pAc-LvRelish-V5 or pAc-IKKβ-FLAG was also co-transfected with pAc-GFP-HA as controls. Forty-eight hours after transfection, cells were harvested and lysed with IP lysis buffer (Pierce, Cat No. 87788, USA) with a Halt Protease Inhibitor Cocktail (Merck, Cat No. 524628, Germany). The 90% of the cell lysis were incubated with agarose affinity gel of anti-HA (Merck, Cat No. A2095, Germany) or anti-V5 Agarose Affinity Gel antibody produced in mouse (Merck, A7345-1ML, Germany). The remaining 10% of cell lysis were used as input controls. All samples were subjected to SDS-PAGE assays. The primary antibodies used in western blotting included rabbit anti-FLAG antibody (Merck, Cat No. F7425, Germany), rabbit anti-V5 antibody (Merck, Cat No. AB3792, Germany) and rabbit anti-HA antibody (Merck, Cat No. H6908, Germany). Anti-rabbit IgG HRP-conjugate (Promega, Cat No. W4011, USA) was used as the secondary antibody.

In order to detect endogenous LvSTING–LvRelish interaction *in vivo*, hemocytes were lysed in IP lysis buffer (Pierce, Cat No. 87788, USA) with Halt Protease Inhibitor Cocktail (Thermo, Cat No. 87786, USA), and then incubated with protein G agarose beads (CST, Cat No. 37478S, USA) coated with anti-LvRelish antibody (Prepared by Genecreate, China) or a normal rabbit IgG antibody (CST, Cat No. 7074S, USA) for 3 hours at 4 °C, and finally were detected by western blotting with anti-LvSTING antibody (Prepared by Genecreate, China). Five percent of the cell lysis was loaded as the input control.

### Dual-luciferase reporter assays

*Drosophila* S2 cells were transfected with reporter gene plasmids, pRL-TK renilla luciferase plasmid (as an internal control), and expression plasmid (pAc-LvRelish-V5, pAc-IKKβ-FLAG and pAc-LvSTING-HA) or empty pAc5.1/V5-His A plasmid (as a control) using the FuGENE Transfection Reagent (Promega, Cat. No. e2311, USA) following the manufacturer’s protocol. At 48 h after transfection, the firefly and renilla luciferase activity was measured following the manufacturer’s protocol. Three replicates were performed for each experiment.

### Double-stranded RNAs synthesis

DsRNAs specifically targeted to the LvSTING, LvIKKβ or LvRelish, were synthesized through *in vitro* transcription *via* T7 RiboMAX Express RNAi System kit (Promega, Cat. no. P1700, USA). DsRNA-GFP (dsGFP) targeting GFP (Genbank accession DQ389577) was used as a control. Primer sequences were listed in **Table 1**.

### Quantitative PCR analysis

Sample gaining, total RNA extraction and quantitative PCR (qPCR) assays were conducted as previously described (18). The expression levels of target genes were determined using the Livak (2-ΔΔCT) method following normalization to *L. vannamei EF-1a* (GU136229). Primer sequences were listed in **Table 1**. Three replicates were performed for each experiment.

### *V. parahaemolyticus* challenge experiments in LvRelish-knockdown shrimp

To investigate whether LvRelish played a protective role against *V. parahaemolyticus*, healthy shrimp were separated into two groups and injected with dsGFP or dsRNA-LvRelish (dsLvRelish). Each shrimp was injected with dsRNA (2 μg/g shrimp). Shrimp were then injected with *V. parahaemolyticus* or PBS after 48 h and maintained in culture flasks for approximately a week following infection. Surviving shrimp numbers were recorded every 4 h.

Another experiment was conducted to monitor the abundance of *V. parahaemolyticus* in LvRelish-knockdown shrimp. Quantitative PCR (qPCR) assays were conducted as previously described [10]. At 12 h after *V. parahaemolyticus* infection, gill tissue samples were collected from each group to extract DNA. The Marine Animals DNA Kit (TIANGEN, Cat. No. DP324, China) was used to extract gill DNA. The number of bacteria in gill tissue samples was quantified through qPCR using the *V. parahaemolyticus* 16S rRNA gene (rDNA, GenBank No. EU660325) with specific primers (**Table 1**) (19). In brief, serial dilutions (108, 107, 106, 105, 104, and 103 copies) of plasmids containing *V. parahaemolyticus* 16S rRNA gene fragments were used to construct the standard curve. The genome copies of *V. parahaemolyticus* in 1 g gill tissue samples were then calculated. Hemocyte RNA was extracted to determine the expression of LvRelish for RNAi efficiency, and to detect the expression of shrimp NF-κB-mediated effector genes (LvALF1, LvLYZ1, LvCRU1, and LvPEN4). Three replicates were performed for each experiment.

### Expression of LvSTING in hemocyte from *V. parahaemolyticus*-challenged shrimp

The expression patterns of LvSTING in the hemocytes from *V. parahaemolyticus*-challenged shrimp were investigated. For immune stimulations assay, the treated groups were injected with *V. parahaemolyticus* solution (1 × 105 CFU), and the control group was injected with PBS solution. Hemocytes of challenged shrimp were sampled at 0, 3, 6, 12, 24, 36, 48 h post injection (hpi), and each sample was collected and pooled from 5 shrimp. Primer sequences were listed in **Table 1**.

### Immunofluorescence assay

Forty-eight hours post dsGFP, dsRNA-LvSTING (dsLvSTING) or dsRNA-LvIKKβ (dsLvIKKβ) injection, shrimp were injected with 50 µl PBS or a suspension of approximately 1 × 105 CFU of *V. parahaemolyticus*. At six hours after *V. parahaemolyticus* infection, shrimp hemocytes were obtained through centrifugation (1000 g for 5* min*) at 25 °C and seeded onto the slides. The hemocytes were fixed with 4% paraformaldehyde, and then were permeabilized with methanol at -20 °C. The slides were blocked using 3% BSA for 1 h at 25 °C and then incubated overnight (at 4°C for approximately 8 h) in a mixture of rabbit anti-LvRelish antibody (Genecreate, China) and mouse anti-β-actin antibody (Merck, Cat. No. A2228, Germany). The hemocytes were then washed with PBS and incubated with the fluorescent antibody (CST, Cat. No. 4412S/8890S, USA) for 1 h at 25 °C in the dark. The hemocytes were washed with PBS and stained with Hoechst 33258 (Yeasen, Cat. No. 40729ES10, China) for 10 min at 25 °C before being washed another six times. The fluorescence was visualized using a confocal laser scanning microscope (Leica, TCS-SP8, Germany). WCIF ImageJ software was used to analyze the colocalization of LvRelish and Hochest-stained nuclei in hemocytes.

### Relish phosphorylation detection

Shrimp hemocytes were harvested at 6 hours post *V. parahaemolyticus* infection and lysed with IP lysis buffer (Pierce, Cat No. 87788, USA) with a Halt Protease Inhibitor Cocktail (Merck, Cat No. 524628, Germany). All samples were subjected to SDS-PAGE assays. The primary antibodies used in western blotting included rabbit anti-pLvRelish antibody (Genecreate, China) and mouse anti-β-actin antibody (Merck, Cat. No. A2228, Germany). Anti-rabbit IgG (H+L) HRP-conjugate (Promega, Cat No. W4011, USA) and Anti-mouse IgG (H+L) HRP-conjugate (Promega, Cat No. W4021, USA) were used as the secondary antibody.

### *V. parahaemolyticus* challenge experiments in shrimp treated with dsIKKβ or dsSTING

To investigate whether LvSTING can activate LvRelish, healthy shrimp were separated into two groups and injected with dsGFP or dsLvSTING. Each shrimp was injected with dsRNA (2 μg/g shrimp). 48 hours post dsRNA injection, shrimp were infected with *V. parahaemolyticus* or PBS. And 12 hours post *V. parahaemolyticus* infection, hemocytes were harvested for qPCR, western blotting and immunofluorescence assay, and gill tissue samples were collected for *V. parahaemolyticus* numbers. Three replicates were performed for each experiment.

To explore whether LvIKKβ participates in LvRelish activation, healthy shrimp were separated into two groups and injected with dsGFP or dsLvRelish. Each shrimp was injected with dsRNA (2 μg/g shrimp). 48 hours post dsRNA injection, shrimp were infected with *V. parahaemolyticus* or PBS. And 12 hours post *V. parahaemolyticus* infection, hemocytes were harvested for qPCR, western blotting and immunofluorescence assay. Gill tissue samples were collected for counting *V. parahaemolyticus* numbers. Three replicates were performed for each experiment.

To prove that the LvSTING**–**LvIKKβ**–**LvRelish**–**AMPs axis plays a protective role against *V. parahaemolyticus*, healthy shrimp were separated into four groups and injected with dsGFP (4 μg/g shrimp), dsLvSTING (2 μg/g shrimp) plus dsGFP (2 μg/g shrimp), dsLvIKKβ (2 μg/g shrimp) plus dsGFP (2 μg/g shrimp), and dsLvSTING (2 μg/g shrimp) plus dsLvIKKβ (2 μg/g shrimp). Shrimp were then injected with *V. parahaemolyticus* or PBS after 48 hours and maintained in culture flasks for approximately a week following infection. Surviving shrimp numbers were recorded every 4 h. And 12 hours post *V. parahaemolyticus* infection, hemocytes were harvested for qPCR, and gill tissue samples were collected for counting *V. parahaemolyticus* numbers. Three replicates were performed for each experiment.

### Statistical analysis

All the data are presented as mean ± SD. Student’s *t* test is used to calculate the comparisons between groups of numerical data. For survival rates, data are subjected to statistical analysis using GraphPad Prism software to generate the Kaplan ± Meier plot (log-rank χ2 test). The following *p* values are considered to be statistically significant: *p < 0.05, **p < 0.01 and ***p < 0.001.

## Results

### LvRelish could defend against *V. parahaemolyticus* infection *via* inducing AMPs

**Figure 1:**
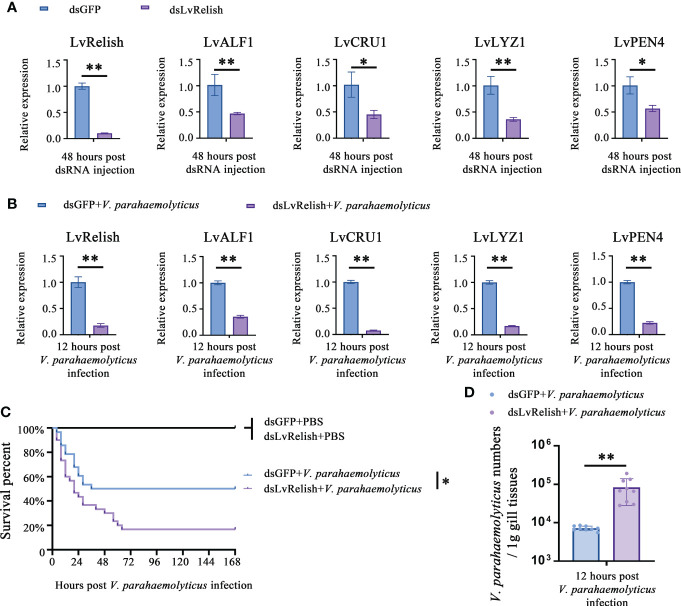
LvRelish defended against *V. parahaemolyticus* infection *via* inducing AMPs. **(A)** Relative expression of LvRelish and AMPs in LvRelish silenced shrimp at 48 hours post dsRNA injection. **(B)** Relative expression of LvRelish and AMPs in LvRelish silenced shrimp at 12 hours post *V. parahaemolyticus* infection. **(C)** Percent survival of LvRelish silenced shrimp after *V. parahaemolyticus* infection. The experiments were performed three times with identical results. Differences between groups were analyzed with Log-rank test using the software of GraphPad Prism 5.0 (*p < 0.05). **(D)** *V. parahaemolyticus* numbers in gill tissues of LvRelish silenced shrimp at 12 hours post *V. parahaemolyticus* infection. One dot represents one sample and the column represents the median of the results. The data **(A, B, D)** was analyzed statistically by student’s T test (*p < 0.05, **p < 0.01).

Shrimp NF-κB pathway is crucial for AMPs expression, and LvRelish is a key transcription factor of NF-κB pathway. In this study, RNAi was performed to investigate the relationship between LvRelish and AMPs. We designed and synthesized dsRNA-LvRelish (dsLvRelish) targeting LvRelish expression, and checked the silencing efficiency of LvRelish at 48 h post dsRNA injection. The injection of dsLvRelish resulted in a significant decrease in LvRelish expression levels down-regulating to ~0.11-fold of the control group (**Figure 1A**), which was sufficient for the following experiments. Accordingly, the expression levels of LvALF1, LvCRU1, LvLYZ1 and LvPEN4 were remarkably down-regulated to ~0.47-fold, ~0.45-fold, ~0.36-fold and ~0.57-fold compared to those of dsGFP group at 48 h post dsRNA injection (**Figure 1A**).

Considering the relationship between LvRelish and AMPs, we were curious about the role played by LvRelish in the host defense against bacterial infection. As shown in **Figure 1B**, the expression of LvRelish in dsLvRelish treated group was down-regulated to ~0.30-fold comparing with the dsGFP group, which meant dsLvRelish was competent for LvRelish knockdown during *V. parahaemolyticus* infection. And the detection of AMPs during *V. parahaemolyticus* infection showed that the expression of AMPs in dsLvRelish injected shrimp were lower than those of the control group (**Figure 1B**). These results suggested that LvRelish could regulate AMPs expressions in both uninfected shrimp (**Figure 1A**) and *V. parahaemolyticus*-infected shrimp (**Figure 1B**).

During *V. parahaemolyticus* infection, the survival rate of dsLvRelish group was much lower than that of dsGFP group (χ2: 8.674, *p* = 0.0340), which indicated that LvRelish silenced shrimp were more sensitive to *V. parahaemolyticus* infection (**Figure 1C**). Besides, the higher numbers of *V. parahaemolyticus* were observed in dsLvRelish group at 12 h post *V. parahaemolyticus* infection (**Figure 1D**) that correlated well with the survival percent recorded in **Figure 1C**, and further confirmed that LvRelish played an antibacterial role in the innate immune response.

### LvSTING triggered AMPs expression *via* interacting with LvRelish *in vitro*

**Figure 2:**
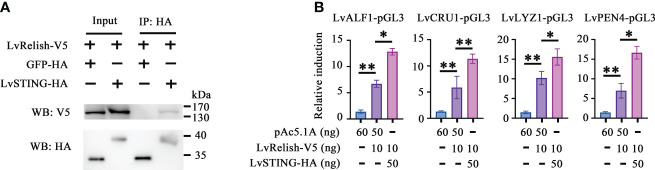
LvSTING triggered AMPs expression *via* interacting with LvRelish *in vitro*. **(A)** The Co-IP assays detecting the interaction between LvSTING and LvRelish. **(B)** The promoter activities of shrimp AMPs were induced by LvRelish with or without LvSTING in S2 cells. The data was analyzed statistically by student’s T test (*p < 0.05, **p < 0.01).

Relish is the vital transcription factor of STING-mediated pathways in silkworm and fruit fly (12, 20), but whether LvSTING participates in Relish regulation is still unclear. In this study, we found that V5 tagged LvRelish was co-immunoprecipitated with HA tagged LvSTING, but no appreciable binding was observed for HA tagged GFP protein (**Figure 2A**), which suggested that LvSTING could interact with LvRelish.

Given the vital role acted by LvRelish in inducing AMPs, the discovery of the association between LvSTING and LvRelish implied that LvSTING might induce AMPs by LvRelish. As **Figure 2B** shown, LvRelish-triggered promoter activities of LvALF1, LvCRU1, LvLYZ1 and LvPEN4, were promoted by the co-expression of LvSTING in S2 cells, which demonstrated that LvSTING could enhance LvRelish-mediated AMPs expression *in vitro*.

### LvSTING prompted LvRelish activation *in vivo*

**Figure 3:**
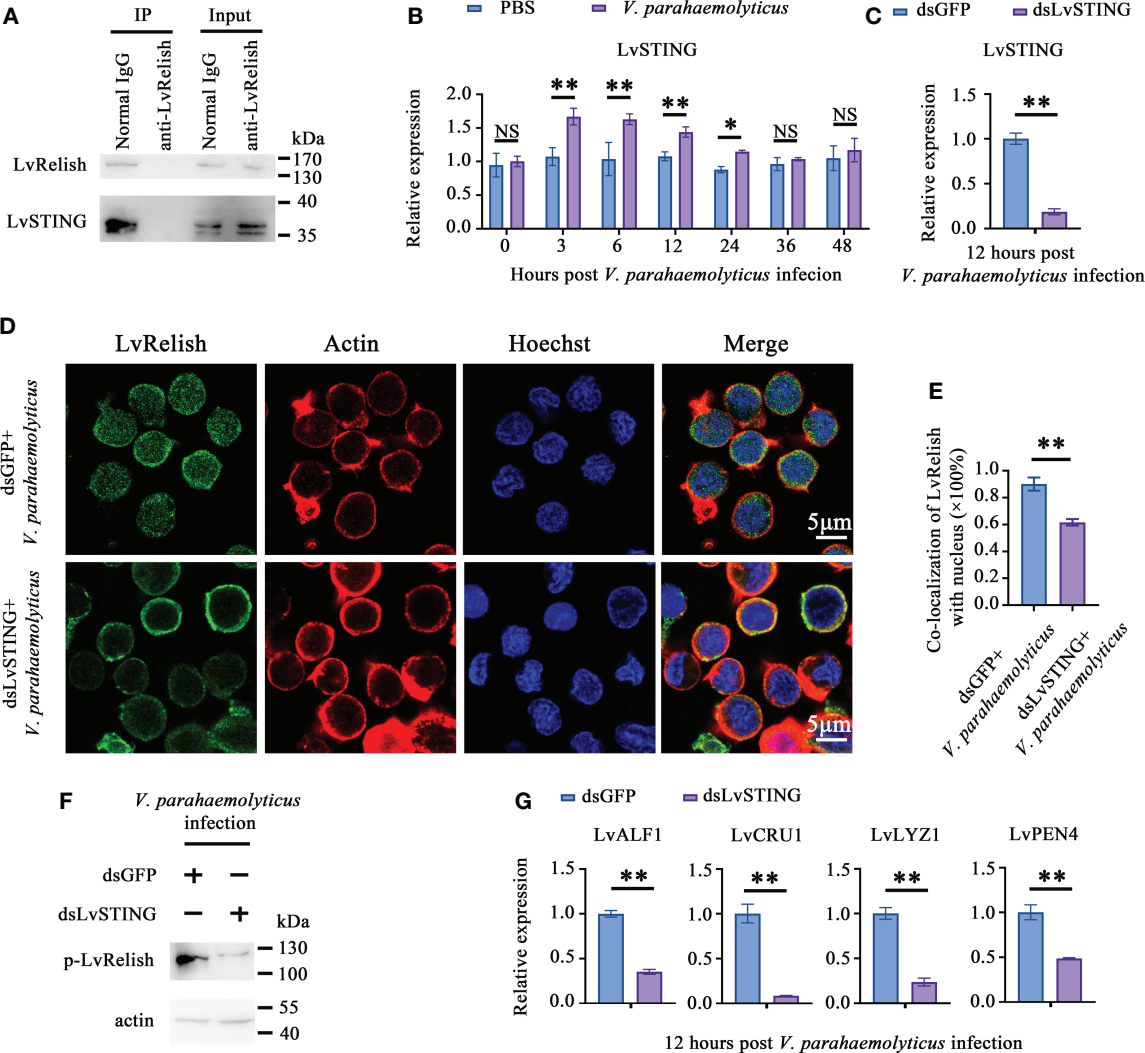
LvSTING prompted LvRelish activation *in vivo*. **(A)** IP assay detecting the interaction between LvSTING and LvRelish *in vivo*. **(B)** Expression profiles of LvSTING in hemocytes from PBS or *V. parahaemolyticus* challenged shrimp. The expression level at each time points were normalized to 0 h post PBS-injected group. **(C)** RNAi efficiency of dsLvSTING in shrimp hemocytes. **(D)** LvRelish nuclear translocation in LvSTING silenced hemocytes infected by *V. parahaemolyticus*. **(E)** Co-localization of LvRelish and Hochest-stained nucleus in hemocytes corresponding to Figure 3D calculated by WCIF ImageJ software. **(F)** The phosphorylation levels of LvRelish in the hemocytes from dsLvSTING or dsGFP treated shrimp during *V. parahaemolyticus* infection. **(G)** The expression of AMPs in the hemocytes of dsLvSTING or dsGFP treated shrimp. The data was analyzed statistically by student’s T test (*p < 0.05, **p < 0.01).

To prove the interaction between LvSTING and LvRelish existed in shrimp hemocyte, IP experiments were performed with LvRelish antibody *in vivo*. IP assays demonstrated that LvRelish could interact with LvSTING in hemocyte (**Figure 3A**). In hemocyte of *V. parahaemolyticus* infected shrimp, LvSTING expressions were up-regulated at 3, 6, 12, 24 hours post *V. parahaemolyticus* infection, indicated that LvSTING also responded to *V. parahaemolyticus* infection in hemocyte (**Figure 3B**). Then, we confirmed the effects of LvSTING on LvRelish activation *in vivo*. As **Figure 3C** shown, dsLvSTING could efficiently suppress LvSTING expression to ~0.19-fold of control. LvRelish has been proved to transfer from cytoplasm into nuclear after *V. parahaemolyticus* infection (21), and LvRelish nuclear location was inhibited by LvSTING knockdown during *V. parahaemolyticus* infection (**Figures 3D, E**). And the phosphorylation of LvRelish was weakened in hemocytes from LvSTING knocked down shrimp (**Figure 3F**), suggesting that LvSTING was involved in activating LvRelish. LvSTING knockdown led to down-regulated the expression of LvALF1, LvCRU1, LvLYZ1 and LvPEN4 during *V. parahaemolyticus* infection (**Figure 3G**), which indicated that LvSTING was involved in AMPs expression in shrimp. To sum up, LvSTING prompted LvRelish phosphorylation and nuclear location to induce AMPs expression.

### LvIKKß was required for AMPs expression and LvRelish activation

**Figure 4:**
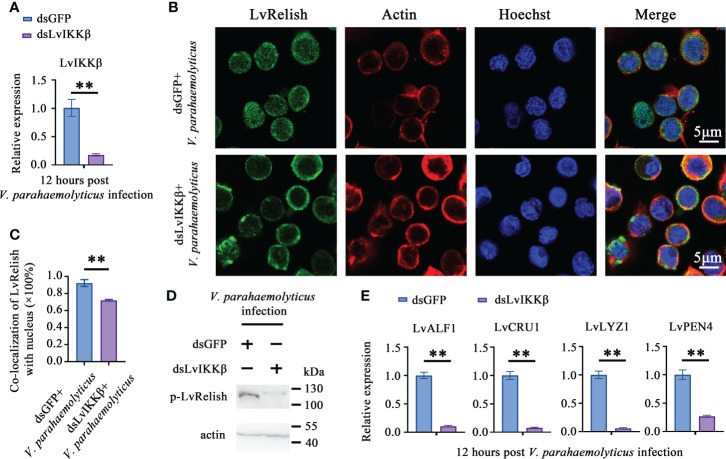
LvIKKß is required for AMPs expression and LvRelish activation. **(A)** RNAi efficiency of dsLvIKKβ in shrimp hemocytes. **(B)** LvRelish nuclear translocation in LvIKKβ silenced hemocytes infected by *V. parahaemolyticus*. **(C)** Co-localization of LvRelish and Hochest-stained nucleus in hemocytes corresponding to Figure 4B calculated by WCIF ImageJ software. **(D)** The phosphorylation levels of LvRelish in the hemocytes of dsLvIKKβ and dsGFP treated shrimp during *V. parahaemolyticus* infection. **(E)** The expression of AMPs in the hemocytes of dsIKKβ or dsGFP treated shrimp. The data was analyzed statistically by student’s T test (**p < 0.01).

IKKβ is the phosphokinase targeting Relish in *Drosophila* IMD pathway (22), but no direct evidence supporting that shrimp IKKβ activates Relish. In this study, dsLvIKKβ was used to inhibit the expression of LvIKKβ (**Figure 4A**), and the effect of LvIKKβ on LvRelish activation was examined. LvRelish subcellular location was observed by immunofluorescence assay. The results showed that LvRelish moved into the nuclear in response to *V. parahaemolyticus* infection, and dsLvIKKβ injection could suppress nuclear import of LvRelish (**Figures 4B, C**). LvRelish phosphorylation levels in hemocytes were significantly reduced by silencing LvIKKβ expression (**Figure 4D**). QPCR performed that dsLvIKKβ inhibited AMPs expression (**Figure 4E**), which is consistent with the AMPs expression changes caused by dsLvRelish or dsLvSTING. The data above indicated that LvIKKβ induced AMPs expression *via* activating LvRelish.

### LvSTING recruited LvIKKβ and LvRelish to synergistically induce AMPs *in vitro*

**Figure 5:**
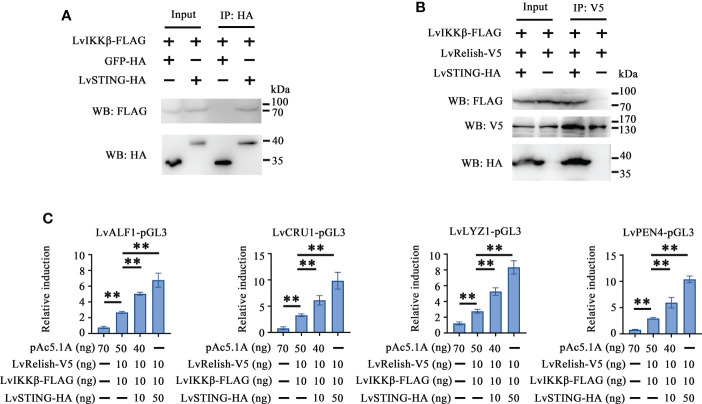
LvSTING recruited LvIKKβ and LvRelish to induce the promoter activities of AMPs *in vitro*. **(A)** The Co-IP assays confirmed the interaction between LvSTING and LvIKKβ. **(B)** The Co-IP assays proved that LvSTING recruited LvRelish and LvIKKβ. **(C)** The promoter activities of shrimp AMPs induced by LvIKKβ and LvRelish could be upregulated by ectopic expression of LvSTING in a dose-dependent manner. The data was analyzed statistically by student’s T test (**p < 0.01).

To explore the effects of LvSTING on LvIKKβ–LvRelish signaling transduction, the interaction between LvSTING and LvIKKβ was examined. The Co-IP demonstrated that FLAG-tagged LvIKKβ interacted with HA-tagged LvSTING but not HA-tagged GFP, which indicated that LvSTING could recruit LvIKKβ (**Figure 5A**). To prove the recruitment functions of LvSTING, FLAG-tagged LvIKKβ and V5-tagged LvRelish were co-expressed with or without HA-tagged LvSTING in S2 cells. As shown in **Figure 5B**, FLAG-tagged LvIKKβ was co-immunoprecipitated with V5-tagged LvRelish with the help of HA-tagged LvSTING, and the absence of LvSTING resulted in less LvIKKβ was co-immunoprecipitated with LvRelish. To confirm the effect of LvSTING on LvIKKβ–LvRelish–AMPs pathway, the dual luciferase assays was performed in S2 cells. The result showed that the promoter activities of shrimp AMPs (LvALF1, LvCRU1, LvLYZ1 and LvPEN4) could be positively regulated by the co-expression of LvIKKβ and LvRelish, which could be further upregulated by ectopic expression of LvSTING in a dose-dependent manner (**Figure 5C**).

### The LvSTING–LvIKKβ–LvRelish–AMPs axis played a protective role against *V. parahaemolyticus*

**Figure 6:**
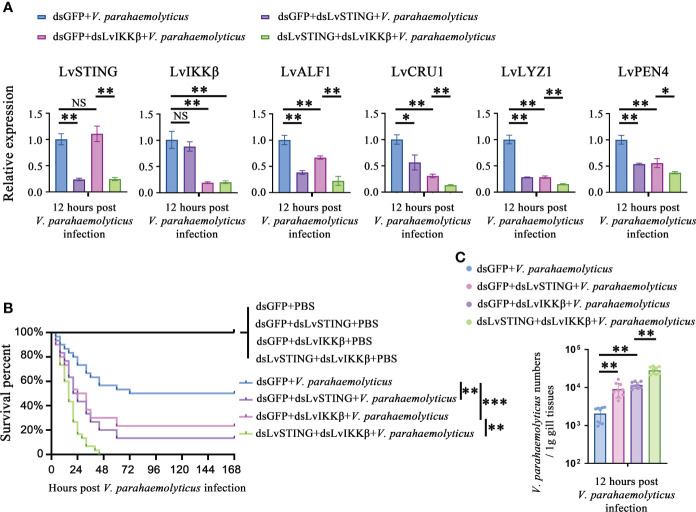
The LvSTING–LvIKKβ–LvRelish–AMPs axis played a protective role against *V. parahaemolyticus.* ***(*A*)*** Relative expression of LvSTING, LvIKKβ and AMPs in the hemocytes from dsLvIKKβ or dsGFP treated shrimp with or without dsLvSTING injection at 12 hours post *V. parahaemolyticus* infection. **(B)** Percent survival of dsLvIKKβ or dsGFP treated shrimp with or without dsLvSTING injection after *V. parahaemolyticus* infection. The experiments were performed three times with identical results. Differences between groups were analyzed with Log-rank test using the software of GraphPad Prism 5.0 (**p < 0.01, ***p < 0.001). **(C)** *V. parahaemolyticus* numbers in gill tissues of dsLvIKKβ or dsGFP treated shrimp with or without dsLvSTING injection at 12 hours post *V. parahaemolyticus* infection. One dot represents one sample and the column represents the median of the results. The data **(A, C)** was analyzed statistically by student’s T test (*p < 0.05, **p < 0.01). ns, no significant difference.

As mentioned above, there was a LvSTING–LvIKKβ–LvRelish–AMPs signaling pathway in shrimp. We were curious about the roles played by the above signaling pathway in the host defense against *V. parahaemolyticus* infection. Double knockdown experiments were done to investigate whether LvSTING regulated the expression of AMPs through LvIKKβ. We observed that LvSTING- or LvIKKβ-knockdown reduced the expression of AMPs during *V. parahaemolyticus* infection. When compared to dsLvIKKβ-injected shrimp, LvSTING and LvIKKβ knockdown combined suppressed the expression of LvALF1 (~0.34-fold), LvCRU1 (~0.43-fold), LvLYZ1 (~0.53-fold) and LvPEN4 (~0.67-fold) (**Figure 6A**). Furthermore, **Figure 6B** showed that knockdown of LvSTING or LvIKKβ increased shrimp susceptibility to *V. parahaemolyticus* infection compared to the dsGFP group (χ2 = 10.32, *p* = 0.0013; χ2 = 5.662, *p* = 0.0173). In addition, when compared to LvIKKβ-silenced shrimp, LvSTING and LvIKKβ knockdown resulted in higher cumulative mortality (χ2 = 9.974, *p* = 0.0016), suggesting that LvSTING improved shrimp resistance to *V. parahaemolyticus* infection *via* LvIKKβ (**Figure 6B**). In agreement with the survival curves, LvSTING- or LvIKKβ-knockdown boosted *V. parahaemolyticus* levels in shrimp gill tissues, and *V. parahaemolyticus* numbers in the dsLvSTING + dsLvIKKβ group were substantially greater than those in the LvIKKβ-silenced alone group (**Figure 6C**). Taken together, these results suggested that LvSTING could trigger an antibacterial response *via* the LvIKKβ**–**LvRelish**–**AMPs pathway during *V. parahaemolyticus* infection.

## Discussion

In the last decade, studies on cytosolic surveillance systems have advanced significantly, highlighting the key role of the cGAS-STING signaling pathway in bacterial infection. In vertebrates, STING has been reported to be triggered by bacterial DNA-activated cGAS or another DNA sensor, IFI16, leading to the production of IFN-I during *Listeria monocytogenes* infection (23). STING is also able to recognize bacteria derived CDNs, including 3’3’-cGAMP, c-di-GMP and c-di-AMP (24–26). Hence, vertebrates STINGs detect a wide range of ligands from bacterial infection, which leads to rapid antibacterial immune responses.

STING can also respond to bacterial infection in invertebrates. *D. melanogaster* STING (DmSTING) senses c-di-GMP and induces a set of AMPs production to control *L. monocytogenes* infection through transcription factor Relish (27). Likewise, *V. parahaemolyticus* infection substantially increased LvSTING expression in intestine and hepatopancreas, demonstrating that LvSTING played a role in innate immunity against bacterial infection (14). In this study, LvSTING expression in hemocytes was shown to be dramatically increased at 3, 6, 12, and 24 hours after *V. parahaemolyticus* infection, indicating that LvSTING was responding to *V. parahaemolyticus* invasion in hemocytes. Because hemocytes are considered as the most essential immune cells in shrimp for pathogen recognition and phagocytic function (28), the rapid increase of LvSTING in hemocytes indicated that it could play a role comparable to DmSTING in innate immune response to bacterial invasion.

Since IFN pathways are not widespread in invertebrates, STING-dependent Relish activation is a critical method for defending against pathogen invasion *via* inducing AMPs expression. A nucleopolyheedrovirus (NPV)-infected *Bombyx mori* cell produces the 2’3’-cGAMP, which binds to *B. mori* STING (BmSTING). Ligand-binding of BmSTING dissociates from the suppressor caspase-8-like protein (BmCasp8L), and triggers BmRelish cleavage by death-related ced-3/Nedd2-like caspase (BmDredd) to induce the expression of AMPs such as BmCecropinA and BmCecropinB (20). In Shrimp, Relish activation causes a rise in the expression of a variety of AMPs, which is one of the most important ways to eliminate germs from the host (29). The four kinds of AMPs from shrimp, such as anti-LPS-factor (ALF), Crustin (CRU), Lysozyme (LYZ) and Penaeidin (PEN), have been identified as Relish-mediated effectors and have broad anti-microbial properties to Gram-positive and Gram-negative bacteria (29–32). Our results showed that LvSTING activated LvRelish *via* LvIKKβ, and induced AMPs including LvALF1, LvCRU1, LvLYZ1 and LvPEN4, playing a protective role against *V. parahaemolyticus* infection. Accumulating evidence suggested that AMPs’ synthesis *via* the STING–Relish cascade could be a powerful antibacterial mechanism of invertebrates.

Despite the fact that transcription of type I IFN genes is the primary antiviral output of STING signaling in mammals, these genes have only been discovered in vertebrates (33). IRF3, the transcription factor that drives to type I IFN transcription after STING activation, is only found in vertebrates and several kinds of invertebrates (6, 34). Therefore, IFN pathways regulated by STING are not widely distributed among invertebrates. NF-κB activation is another downstream consequence of STING-mediated signaling (35). Key components of NF-κB pathways are conserved in vertebrates and invertebrates, and NF-κB and IκB homologs have been identified in most animal lineages (36). Although STING-mediated NF-κB activation has been discovered in the insects including *B. mori* (20) and *D. melanogaster* (12), but not clear in other species of invertebrates. This study demonstrated that STING-mediated Relish activation happens in shrimp and protects them from bacterial invasion. The present work could contribute to the understanding of how STING-mediated NF-κB activation occurs in vertebrates.

In summary, we described an innate immune pathway against *V. parahaemolyticus*. LvSTING responded to *V. parahaemolyticus* invasion, then recruited LvIKKβ and LvRelish, leading to LvRelish phosphorylation and nuclear translocation. The activated LvRelish translocated into nuclear and induced AMPs expression, which were the crucial antibacterial effectors to kill *V. parahaemolyticus.* We identified the LvSTING–LvIKKβ–LvRelish–AMPs signaling pathway against *V. parahaemolyticus*, which helped us learn more about LvSTING’s role in shrimp and gave us some insight into disease resistance breeding.

## Data availability statement

The original contributions presented in the study are included in the article/Supplementary Material. Further inquiries can be directed to the corresponding authors.

## Author contributions

HL: conceived, designed and performed the experiments, and analyzed data, as well as wrote the draft manuscript. QL and SW: performed the experiments and analyzed data. JH: conceived and designed the experiments, and acquired finding. CL: conceived and designed the experiments, acquired finding, and was responsible for forming the hypothesis, project development, data coordination, and writing, finalizing, and submitting the manuscript. All authors discussed the results and approved the final version.

## Funding

This research was supported by National Natural Science Foundation of China (31930113/32173000/32022085), Natural Science Foundation of Guangdong Province (2021A1515010747), Science and Technology Planning Project of Guangzhou City (202102020354), Independent Research and Development Projects of Maoming Laboratory (2021ZZ007/2021TDQD004), Southern Marine Science and Engineering Guangdong Laboratory (Zhuhai) (SML2021SP301), and the Fundamental Research Funds for the Central Universities, Sun Yat-sen University (22lglj05). The funders had no role in study design, data collection and analysis, decision to publish, or preparation of the manuscript.

## Conflict of interest

The authors declare that the research was conducted in the absence of any commercial or financial relationships that could be construed as a potential conflict of interest.

## Publisher’s note

All claims expressed in this article are solely those of the authors and do not necessarily represent those of their affiliated organizations, or those of the publisher, the editors and the reviewers. Any product that may be evaluated in this article, or claim that may be made by its manufacturer, is not guaranteed or endorsed by the publisher.

## References

[1] Food and Agriculture Organization of the United Nations, Rome, Italy. Fishery and aquaculture statistics 2019. (2021).

[2] De Schryver P Defoirdt T Sorgeloos P. Early mortality syndrome outbreaks: a microbial management issue in shrimp farming? PLoS Pathog (2014) 10(4):e1003919. doi: 10.1371/journal.ppat.1003919 24763380 PMC3999206

[3] Chandrakala N Priya S. Vibriosis in shrimp aquaculture a review. Int J Sci Res Sci Eng Tech (2017) 3(2):27–33

[4] Huang Z Zeng S Xiong J Hou D Zhou R Xing C. Microecological koch's postulates reveal that intestinal microbiota dysbiosis contributes to shrimp white feces syndrome. Microbiome (2020) 8(1):32. doi: 10.1186/s40168-020-00802-3 32156316 PMC7065354

[5] Barber GN. Cytoplasmic DNA innate immune pathways. Immunol Rev (2011) 243(1):99–108. doi: 10.1111/j.1600-065X.2011.01051.x 21884170

[6] Wu X Wu FH Wang X Wang L Siedow JN Zhang W. Molecular evolutionary and structural analysis of the cytosolic DNA sensor cGAS and STING. Nucleic Acids Res (2014) 42(13):8243–57. doi: 10.1093/nar/gku569 PMC4117786 24981511

[7] Gui X Yang H Li T Tan X Shi P Li M. Autophagy induction *via* STING trafficking is a primordial function of the cGAS pathway. Nature (2019) 567(7747):262–6. doi: 10.1038/s41586-019-1006-9 PMC9417302 30842662

[8] Morehouse BR Govande AA Millman A Keszei AFA Lowey B Ofir G. STING cyclic dinucleotide sensing originated in bacteria. Nature (2020) 586(7829):429–33. doi: 10.1038/s41586-020-2719-5 PMC7572726 32877915

[9] Slavik KM Morehouse BR Ragucci AE Zhou W Ai X Chen Y. cGAS-like receptors sense RNA and control 3'2'-cGAMP signalling in drosophila. Nature (2021) 597(7874):109–13. doi: 10.1038/s41586-021-03743-5 PMC8410604 34261127

[10] Holleufer A Winther KG Gad HH Ai X Chen Y Li L. Two cGAS-like receptors induce antiviral immunity in drosophila. Nature (2021) 597(7874):114–8. doi: 10.1038/s41586-021-03800-z 34261128

[11] Cai H Holleufer A Simonsen B Schneider J Lemoine A Gad HH. 2'3'-cGAMP triggers a STING- and NF-κB-dependent broad antiviral response in drosophila. Sci Signal (2020) 13(660):eabc4537. doi: 10.1126/scisignal.abc4537 33262294

[12] Goto A Okado K Martins N Cai H Barbier V Lamiable O. The kinase IKKβ regulates a STING- and NF-κB-Dependent antiviral response pathway in drosophila. Immunity (2018) 49(2):225–34.e4. doi: 10.1016/j.immuni.2018.07.013 30119996 PMC6267954

[13] Li S Yang F Wang F Lv X Li F. An invertebrate gene encoding a Mab21-containing protein involves in antiviral response through regulating the STING pathway. Dev Comp Immunol (2021) 121:104101. doi: 10.1016/j.dci.2021.104101 33862098

[14] Li H Wang S Lu K Yin B Xiao B Li S. An invertebrate STING from shrimp activates an innate immune defense against bacterial infection. FEBS Lett (2017) 591(7):1010–7. doi: 10.1002/1873-3468.12607 28236646

[15] Li H Yin B Wang S Fu Q Xiao B Lǚ K. RNAi screening identifies a new toll from shrimp litopenaeus vannamei that restricts WSSV infection through activating dorsal to induce antimicrobial peptides. PLoS Pathog (2018) 14(9):e1007109. doi: 10.1371/journal.ppat.1007109 30256850 PMC6175524

[16] Wang S Li H Zhu P Fu Q Yin B Li Q. MAPKKK15 gene from shrimp litopenaeus vannamei is transcribed in larva development stages and contributes to WSSV pathogenesis. Aquaculture (2021) 534:736324. doi: 10.1016/j.aquaculture.2020.736324

[17] Wang S Li H Weng S Li C He J. White spot syndrome virus establishes a novel IE1/JNK/c-jun positive feedback loop to drive replication. iScience (2020) 23(1):100752. doi: 10.1016/j.isci.2019.100752 31884168 PMC6941876

[18] Li H Wang S Chen Y Lǚ K Yin B Li S. Identification of two p53 isoforms from litopenaeus vannamei and their interaction with NF-κB to induce distinct immune response. Sci Rep (2017) 7(1):1–13. doi: 10.1038/srep45821 28361937 PMC5374463

[19] Huang Z Chen Y Weng S Lu X Zhong L Fan W. Multiple bacteria species were involved in hepatopancreas necrosis syndrome (HPNS) of litopenaeus vannamei. Acta Scientiarum Naturalium Universitatis SunYatseni (2016) 55(1):1–11.

[20] Hua X Li B Song L Hu C Li X Wang D. Stimulator of interferon genes (STING) provides insect antiviral immunity by promoting dredd caspase-mediated NF-kappaB activation. J Biol Chem (2018) 293(30):11878–90. doi: 10.1074/jbc.RA117.000194 PMC6066306 29875158

[21] Wang S Li H Chen R Jiang X He J Li C. TAK1 confers antibacterial protection through mediating the activation of MAPK and NF-κB pathways in shrimp. Fish Shellfish Immunol (2022) 123:248–56. doi: 10.1016/j.fsi.2022.03.008 35301113

[22] Stöven S Silverman N Junell A Hedengren-Olcott M Erturk D Engström Y. Caspase-mediated processing of the drosophila NF-κB factor relish. Proc Natl Acad Sci (2003) 100(10):5991–6. doi: 10.1073/pnas.1035902100 PMC156314 12732719

[23] Hansen K Prabakaran T Laustsen A Jørgensen SE Rahbæk SH Jensen SB. Listeria monocytogenes induces IFNβ expression through an IFI16-, cGAS- and STING-dependent pathway. EMBO J (2014) 33(15):1654–66. doi: 10.15252/embj.201488029 PMC4194099 24970844

[24] Romling U Galperin MY Gomelsky M. Cyclic di-GMP: the first 25 years of a universal bacterial second messenger. Microbiol Mol Biol Rev MMBR (2013) 77(1):1–52. doi: 10.1128/MMBR.00043-12 23471616 PMC3591986

[25] Witte CE Whiteley AT Burke TP Sauer JD Portnoy DA Woodward JJ. Cyclic di-AMP is critical for listeria monocytogenes growth, cell wall homeostasis, and establishment of infection. mBio (2013) 4(3):e00282–13. doi: 10.1128/mBio.00282-13 PMC3663569 23716572

[26] Davies BW Bogard RW Young TS Mekalanos JJ. Coordinated regulation of accessory genetic elements produces cyclic di-nucleotides for v. cholerae virulence. Cell (2012) 149(2):358–70. doi: 10.1016/j.cell.2012.01.053 PMC3620040 22500802

[27] Martin M Hiroyasu A Guzman RM Roberts SA Goodman AG. Analysis of drosophila STING reveals an evolutionarily conserved antimicrobial function. Cell Rep (2018) 23(12):3537–50.e6. doi: 10.1016/j.celrep.2018.05.029 29924997 PMC6114933

[28] Tassanakajon A Piti A Kunlaya S Premruethai S. Cationic antimicrobial peptides in penaeid shrimp. Mar Biotechnol (2011) 13(4):639–57. doi: 10.1007/s10126-011-9381-8 21533916

[29] Li C Wang S He J. The two NF-κB pathways regulating bacterial and WSSV infection of shrimp. Front Immunol (2019) 10:1785. doi: 10.3389/fimmu.2019.01785 31417561 PMC6683665

[30] Huang X Yin Z Liao J Wang P Yang L Ai H. Identification and functional study of a shrimp relish homologue. Fish Shellfish Immunol (2009) 27(2):230–8. doi: 10.1016/j.fsi.2009.05.003 19463956

[31] Aweya J Zheng Z Zheng X Yao D Zhang Y. The expanding repertoire of immune-related molecules with antimicrobial activity in penaeid shrimps: a review. Rev Aquacult (2021) 13(4):1907–37. doi: 10.1111/raq.12551

[32] Lin M Hui C Chen J Wu J. The antimicrobial peptide, shrimp anti-lipopolysaccharide factor (SALF), inhibits proinflammatory cytokine expressions through the MAPK and NF-κB pathways in trichomonas vaginalis adherent to HeLa cells. Peptides (2012) 38(2):197–207. doi: 10.1016/j.peptides.2012.10.003 23088922

[33] Gan Z Chen SN Huang B Hou J Nie P. Intronless and intron-containing type I IFN genes coexist in amphibian xenopus tropicalis: Insights into the origin and evolution of type I IFNs in vertebrates. Dev Comp Immunol (2017) 67:166–76. doi: 10.1016/j.dci.2016.10.007 27780747

[34] Lu M Yang C Li M Yi Q Lu G Wu Y. A conserved interferon regulation factor 1 (IRF-1) from pacific oyster crassostrea gigas functioned as an activator of IFN pathway. Fish Shellfish Immunol (2018) 76:68–77. doi: 10.1016/j.fsi.2018.02.024 29458094

[35] Ishikawa H Barber GN. STING is an endoplasmic reticulum adaptor that facilitates innate immune signalling. Nature (2008) 455(7213):674–8. doi: 10.1038/nature07317 PMC2804933 18724357

[36] Margolis SR Wilson SC Vance RE. Evolutionary origins of cGAS-STING signaling. Trends Immunol (2017) 38(10):733–43. doi: 10.1016/j.it.2017.03.004 28416447

